# Using HPLC with In-Column Derivatization to Authenticate Coffee Samples

**DOI:** 10.3390/molecules28041651

**Published:** 2023-02-08

**Authors:** Corey W. Manwaring, Jake A. Cravino, Margi Patel, Jonathan G. H. Stathakis, Arianne Soliven, Thirada Suktham, Ross Andrew Shalliker

**Affiliations:** 1 School of Science, Western Sydney University, South Parramatta, NSW 2150, Australia; 2 Ubiquinox, Parramatta, NSW 2151, Australia; 3 Australian Centre of Research on Separation Science, School of Science, Western Sydney University, South Parramatta, NSW 2214, Australia; 4 Avantor, 1 Markham Centre, Station Rd, Theale, Reading Berkshire RG7 4PE, UK

**Keywords:** coffee, antioxidants, CUPRAC, HPLC, postcolumn derivatization, in-column derivatization, authentication

## Abstract

Coffee is one of the world’s most popular beverages, with the global coffee capsule market worth over USD 4 billion and growing. The incidence of coffee fraud is estimated to be up to one in five coffees being contaminated with cheaper blends of coffee. Given the worsening extent of climate change, coffee crop yields are harder to maintain, while demand is increasing. The 2021 Brazil frost delaying or destroying many coffee crops is an example. Hence, the incidence of coffee fraud is expected to increase, and as the market becomes more complex, there needs to be faster, easier, and more robust means of real-time coffee authentication. In this study, we propose the use of novel approaches to postcolumn derivatization (termed herein as in-column derivatization) to visualize the antioxidant profiles of coffee samples, to be later used as indicators for authentication purposes. We propose three simple mathematical similarity metrics for the real-time identification of unknown coffee samples from a sample library. Using the CUPRAC assay, and these metrics, we demonstrate the capabilities of the technique to identify unknown coffee samples from within our library of thirty.

## 1. Introduction

Coffee is consumed worldwide for its flavor, nootropic effects, and health benefits [1], with 6.3 million tons produced [2] and 655 billion cups drunk every year [3]. The coffee bean is produced by the plants *Coffea arabica* (Arabica) and *Coffea canephora* (Robusta), which are grown in over 50 developing tropical and subtropical countries around the world [2,4].

With coffee being widely consumed, consumer-ready coffee capsules have become highly prevalent. This format ensures that users can consistently serve coffee without requiring ‘barista’ expertise. Some of the major capsule brands include Nespresso, Lavazza, Starbucks, Keurig, Gloria Jean’s Coffees, and L’Or. The demand for these fast, consistent, and quality coffee capsules has seen the global coffee capsule market grow to USD 4.06 billion in 2020, with predictions to grow to USD 5 billion by 2025 [5]

Coffee production is a highly lucrative business, so there are numerous instances in which the quality of the coffee, safety, and integrity have often been sacrificed by fraudulent practices. Coffee fraud can occur at various points, either within the supply chain or potentially by the growers, producers, and suppliers themselves to reduce costs and maximize profits by intentionally misleading customers [2]. It is a lucrative business, and there are records of coffee fraud dating back as far as the 19th century [4]. Coffee supply chains necessitate numerous participants and are generally opaque [6], providing those so inclined with plenty of opportunity for fraudulent practices. Climate change is harming production yields, providing an impetus to increase fraudulent practices as profit margins become harder to maintain [7].

One form of fraud is through the cutting of coffee with inferior substances. This can include using cheaper and/or defective beans and cutting with foreign matter, such as husks, stems, soybeans, maize, barley, and brown sugar [8]. In these instances, customers are receiving an inferior product, and they are at risk of adverse health effects, or allergic reactions, from consuming unknown foreign matter [6]. Thus, it is desirable to have a process or test that provides for product authentication, which is the purpose of our present research.

Authentication of the coffee can be achieved by developing a test that establishes a chemical fingerprint or, more accurately, a chemical signature of the sample, which can then be used to characterize the sample according to unique features, either through data mining or chemometric tools [9]. Food fingerprinting techniques for authentication and quality control have proven useful in traceability and have been used extensively for various foods [10,11,12,13].

However, as adulteration practices become more complex, and as the market becomes saturated with the knowledge of authentication techniques, they become easier to deceive [14,15]. Hence, there has been a trend toward developing increasingly complex authentication methods [15], incorporating complex analytical tools, such as high-performance liquid chromatography (HPLC) with mass spectral (MS) detection, nuclear magnetic resonance (NMR) spectroscopy, and isotopic techniques [16]. While successful, they are often too complex and expensive to have widespread use in real-world applications that require rapid, simple, and inexpensive testing to effectively track fraud [12,17]. HPLC, however, utilizing a simple detection process such as UV detection coupled with a chemometric approach to the analysis of the data, is a relatively cheap and simple process that can be used to provide a level of authentication of the food product [18]. Examples of this include the use of nontargeted HPLC-UV fingerprinting techniques using chemometrics [6,19,20]. Recent studies have shown that chromatographic-based methods can produce fingerprints with high discrimination [6,20]. However, UV fingerprinting has become prominent in the industry, particularly when coupled with chromatography [16], and combined with its relative simplicity, it is suspected to be easy to fool. Furthermore, a great deal of repetitious analysis is required for statistical validity. Therefore, what is needed is a technique that is simple in its application but complex in its chemistry and difficult to evade.

The use of HPLC with in-column derivatization (ICD) protocols (an efficient form of postcolumn derivatization), as introduced in the first part of this three-part series, provides a means to separate the components in a complex sample and then selectively detect certain types of compounds within the sample. In the study reported here, the ICD process is utilized for the purpose of detecting antioxidants in the sample, with reduction potentials less than ~0.6 V, by using the well-known CUPRAC reagent. As a result, the nature of the coffee sample is related to the antioxidant content increasing the degree of difficulty required to falsify the analytical outcome of testing since those involved in fraud need to have knowledge of the antioxidants, their quantities, and a means of visualizing their presence. After the chromatographic antioxidant profile for each coffee is revealed, simple mathematical processes can be utilized to effectively differentiate different types of coffee. The work reported here demonstrates the effectiveness of high-resolution separations using in-column derivatization processes to establish the chemical signatures of the coffee samples.

## 2. Experimental Section

### 2.1. Chemicals and Coffee Capsules

Ultrapure Milli-Q water (at 18.2 MΩcm) was prepared in-house. All reagents were of analytical-reagent-grade quality and purchased from Sigma-Aldrich (Castle Hill, NSW, Australia), including formic acid (100%), copper (II) chloride, ammonium acetate, neocuproine (1,2-dimethyl-1,10-phenanthroline), and HPLC-grade methanol.

Coffee capsules were purchased from the local market. Important details of each coffee pertaining to its characteristics are noted in Table 1.

### 2.2. Preparation of Samples and Reagents

The CUPRAC reagent was prepared daily by combining 10 mM of copper (II) chloride solution, 7.5 mM of neocuproine solution, and 1 M of ammonium acetate solution at a 1:1:1 ratio [21].

Coffee samples were prepared from capsules as a 30 mL shot. Two samples of coffee were prepared for each analysis, with the first being discarded and the second taken for analysis. This process was used to clean the machine between coffee preparations. Once prepared, each coffee was cooled to room temperature and subsequently filtered through a 0.2 µm filter prior to injection. No other sample preparation was undertaken. All coffee samples were prepared using Milli-Q water.

### 2.3. Instrumentation and Chromatographic Columns

All separations were undertaken using a Shimadzu (Rydalmere, Australia) HPLC system with a Shimadzu SIL-10 CE vp autoinjector, a Shimadzu SCL-10A vp controller, a Shimadzu LC-20 CE pump, a Shimadzu FCV-10AL vp switching valve, and a vp diode array detector. A Shimadzu LC-10AT vp pump was used to deliver the CUPRAC reagent, and a Phenomenex (Lane Cove, Australia) degasex DG4400 degasser.

The column used in this work was an Avantor^®^ ACE^®^ C18 (100 mm × 4.6 mm) packed with 5 µm particles. This column was fitted with a specially made ‘in-column derivatization’ end fitting (Chromaspeed Pty Ltd., Tonsley, Australia).

### 2.4. Separation Conditions

All analyses were undertaken using gradient elution. Mobile phase A was 100% Milli-Q water; mobile phase B was 100% methanol. Both phases were acidified with 0.1% wt/vol formic acid. The flow rate was 1 mL/minute, with a linear gradient change at a rate of 2.5% per minute. At 100% B, the composition was held for 2 min prior to returning to initial conditions in 2 min. The column was held at initial conditions for 10 min to allow the column bed to re-equilibrate prior to injection of the next sample. The derivatization reagent was added to the flow stream using an ICD insert at the outlet of the HPLC column. The delivery flow rate was 0.3 mL/minute. No mixing loop or any other form of mixer was employed.

Sample injection volumes were 10 µL. The resulting data were collected at 450 nm at a rate of 1.5625 Hz.

## 3. Results and Discussions

### 3.1. In-Column Derivatization

Postcolumn derivatization (PCD) protocols date back to almost the beginning of the HPLC era of chromatography. They have, for example, been the mainstay of amino acid analysis since the 1980s [22,23]. Despite their widespread application leading into the 1990s and the many virtues associated with the advantages of yielding a selective detection process, postcolumn derivatization protocols have not kept pace with advances in separation science. In particular, there are few reports of PCD processes utilizing UHPLC columns since the requirement to add mixing coils after the column greatly decreases the separation performance. Hence, separations involving PCD processes are largely restricted to the older-style HPLC columns with large void volumes [24]; otherwise, the separation performance is sacrificed. 

**Figure 1:**
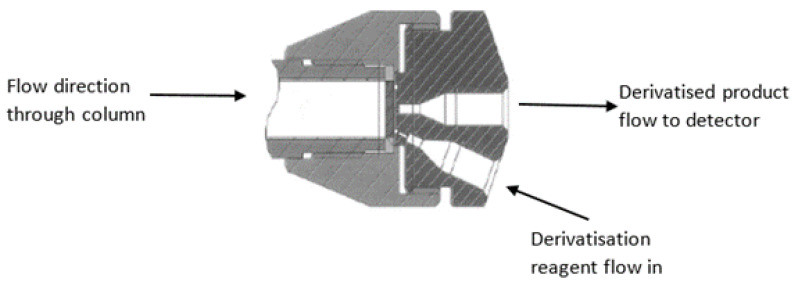
Illustration of the ICD column fitting used for in-column derivatization purposes.

Recently, however, we have developed a new style of PCD process, which negates the need to add additional postcolumn reaction loops and alike. This new style utilizes a novel outlet fitting on the HPLC column that enables the postcolumn reaction to occur inside the column itself (See Figure 1), rather than within a reaction loop, in a postcolumn style. This new form of postcolumn derivatization has been referred to as ‘in-column derivatization’, or ICD, since the reaction occurs in a fitting located at the end of the column. We discussed this new concept in derivatization assays at length in Part 1 of this series. In this second part, we highlight the benefit of employing such a highly efficient separation and detection process for enabling highly detailed chromatographic data that can be used for developing chemical signatures in complex samples. 

**Figure 2:**
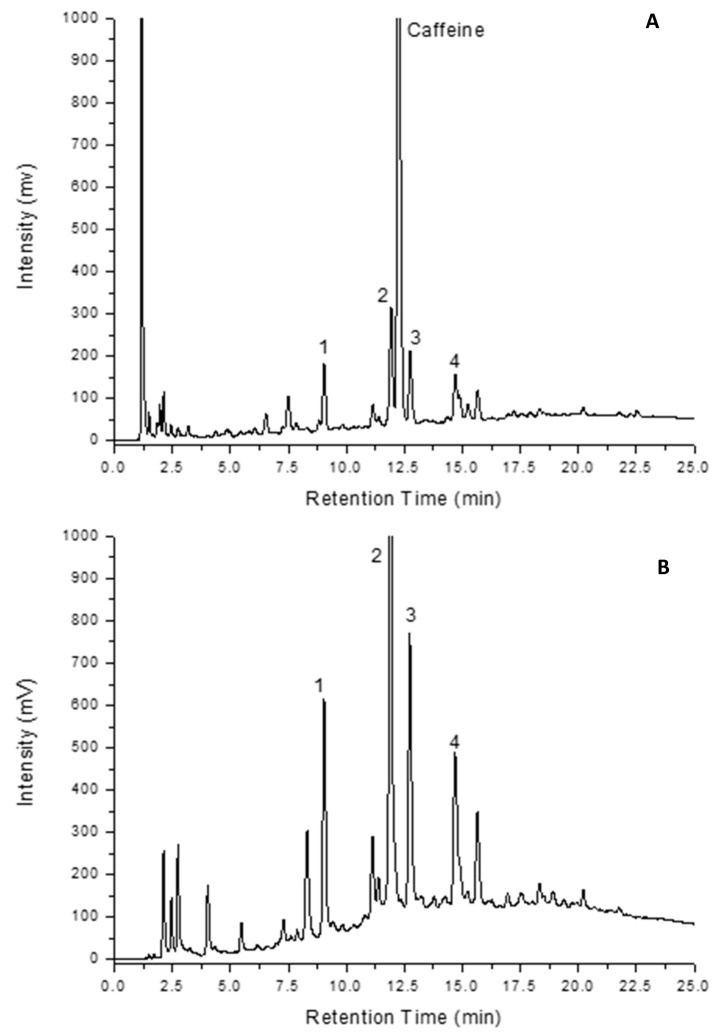
(**A**) UV chromatographic profile of a Ristretto coffee sample at spectral maximum wavelength, and (**B**) ICD chromatographic profile of the same coffee sample at 450 nm.

As an example of the separation performance with and without the derivatization process, two chromatographic profiles of the coffee are illustrated in Figure 2. These separations were obtained on a 100 mm × 4.6 mm C18 column packed with 5 µm particles. While not a UHPLC column, the use of this type of column would ordinarily be out of the scope for applications requiring efficient separations involving PCD protocols [24,25]. Note that both the conventional UV separation and the ICD separation were obtained on the same column fitted with the ICD end-fitting shown in Figure 1. In the case of application in conventional mode, a single port insert can be used instead of the 2-port insert illustrated. Figure 2a is the UV chromatographic profile of a Ristretto coffee sample, while Figure 2b is the ICD chromatographic profile of the same coffee sample. There is virtually no change in the efficiency of the separation when the ICD process is utilized. Four peaks have been labeled in each of these chromatograms for the purpose of reference between each chromatographic profile. Note that caffeine is not reactive to the CUPRAC reagent and, hence, is absent in the ICD profile (between peaks 2 and 3). There are two very important features that are apparent from these profiles: (1) the derivatization process yields information that is distinctly different from that of the UV detection response, and (2) the sensitivity in detection for the antioxidants is increased when utilizing the ICD process (note that signal intensity axes are the same in Figure 2a,b). The combined effects of the high resolution and high sensitivity of the selective detection process provides for an information-rich assay that enables antioxidant indicators to be utilized for the purpose of establishing a chemical signature, which will be developed and discussed throughout this paper.

### 3.2. Coffee Samples

In this study, we analyzed 30 coffee samples in the consumer-ready form of the capsule, and these were sourced from the local market (with the exception of four that were obtained directly from Thailand). Each coffee for analysis was prepared in a standard manner, i.e., as a 30 mL shot, irrespective of the different mass of coffee in each sample, or the style of coffee according to the label claim. As a protocol for yielding a chemical signature, our aim was to have a standardized approach to testing and not to prepare a coffee for the purpose of optimum flavor. The information in Table 1 details the characteristics of each of the coffee samples tested in this work, which includes notes from the manufacturer with respect to tasting. The L’Or Ristretto coffee was chosen as a reference coffee sample (used to ensure proper adherence to operational aspects of the testing). The coffees selected for this study included eleven from the L’Or range, chosen to test whether it was possible to discriminate between differing coffees within a single brand, four coffees from Vittoria, three from Daley Street, six from Starbucks, four from Thailand, three Woolworths Home-brand coffees, and one Moccona coffee. Aside from the differing manufacturers, there were a variety of differing flavors in the selection. 

The mass of coffee contained in each capsule was also measured (Table 1).

### 3.3. Coffee Assays, Data Treatment, and Analysis

#### 3.3.1. Separation

All coffee samples were analyzed as prepared, except that each was filtered through a 0.2 µm filter prior to injection. The sample injection volume was 10 µL. While it may have been possible to undertake a process of defining a chemical signature based on data derived from the UV chromatographic response, our objective in this work was to make it more difficult for counterfeiters to falsify a chemical signature. In that regard, using an assay that actively visualizes specific or rather selective components of the sample, rather than a generalized response, was more promising, as the counterfeiters would need to have a greater understanding of the nature of the coffee and the means to visualize the outcome. This greatly increases the complexity of the process required to falsify or interfere with a sample’s chemical signature. Furthermore, the process of selective detection allowed for the assignment of indicator compounds (antioxidants) that had a distinct profile across most of the coffees tested and this greatly simplified the assessment of the detection data. In the more complex UV chromatograms, it is more difficult to assess visually the key aspects of the chromatogram that would lead to a unique identification.

**Figure 3:**
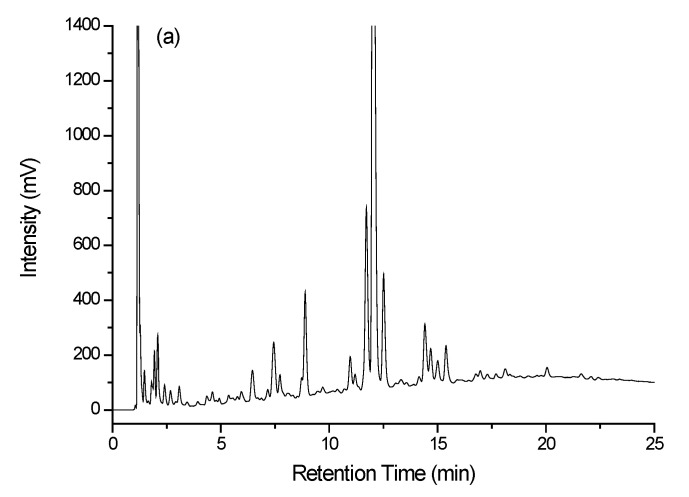

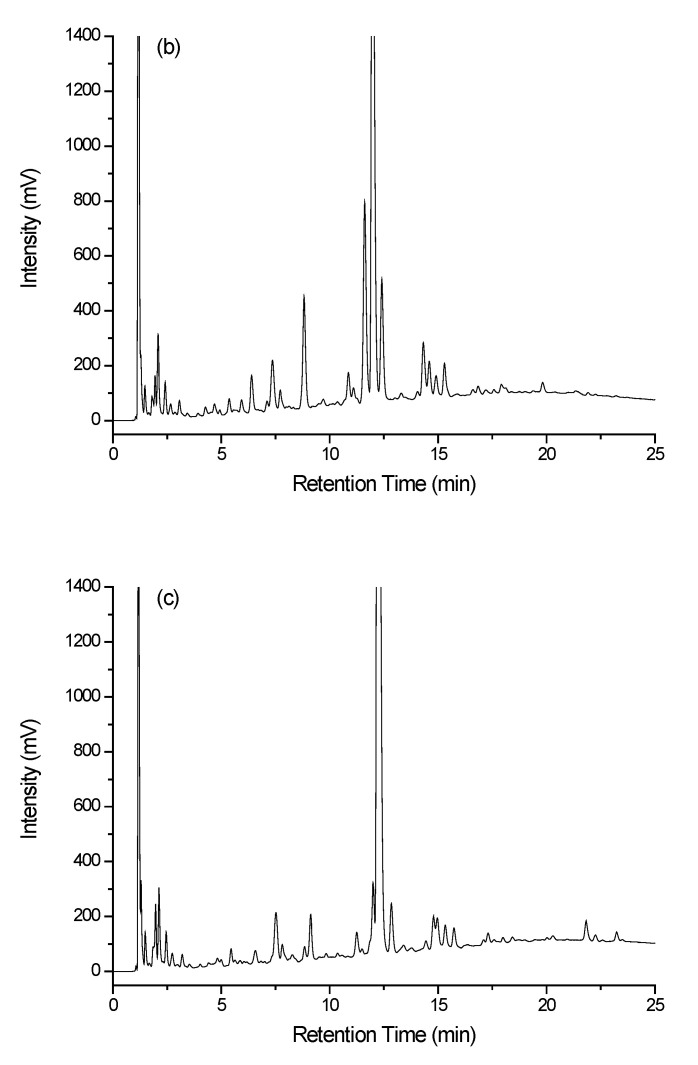
Chromatograms of coffee samples recorded using UV detection at 254 nm. (**a**) Ristretto, (**b**) Profondo, and (**c**) Onyx 12.

The chromatograms illustrated in Figure 3a–c are the UV detection profiles for the (a) Ristretto sample, (b) the Profondo sample, and (c) the Onyx sample, each from the L’Or brand. The UV chromatographic responses for the Ristretto and Profondo coffees are very similar, which ultimately makes it difficult to readily visualize with certainty the differences between these coffees. The chromatogram for the Onyx 12 coffee is, however, more readily distinguishable from those of the Ristretto and the Profondo.

**Figure 4:**
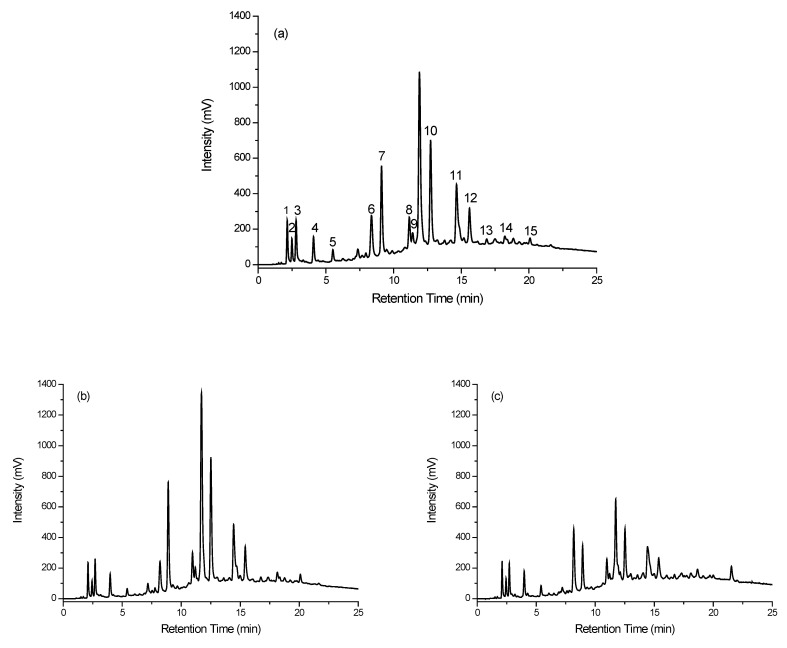
Chromatograms of coffee samples recorded using ICD and detection at 450 nm. (**a**) Ristretto, (**b**) Profondo, and (**c**) Onyx 12.

The chromatographic profiles illustrated in Figure 4a–c are the ICD antioxidant profiles for the same three coffees in Figure 3a–c, respectively. Again, there are similarities between the chromatographic profiles of these three coffees. However, there are subtle differences that can be exploited to yield signatures for these coffees, which is aided by a general reduction in the sample complexity through the targeted analysis of the antioxidants. To differentiate the different coffee samples, 15 indicator compounds were selected (note, we refer to these as indicators, since the selected compounds show a distinct reaction to the antioxidant assay—CUPRAC reagent). These indicators were present in almost every coffee sample tested, but their concentrations differed and, perhaps more importantly, the ratio of the certain groups of indicators depended on the specific coffee sample. The selected indicators are noted in the chromatographic profile shown in Figure 4a—the Ristretto coffee sample. The retention times of these peaks are given in Table 2.

#### 3.3.2. Data Treatment

**Figure 5:**
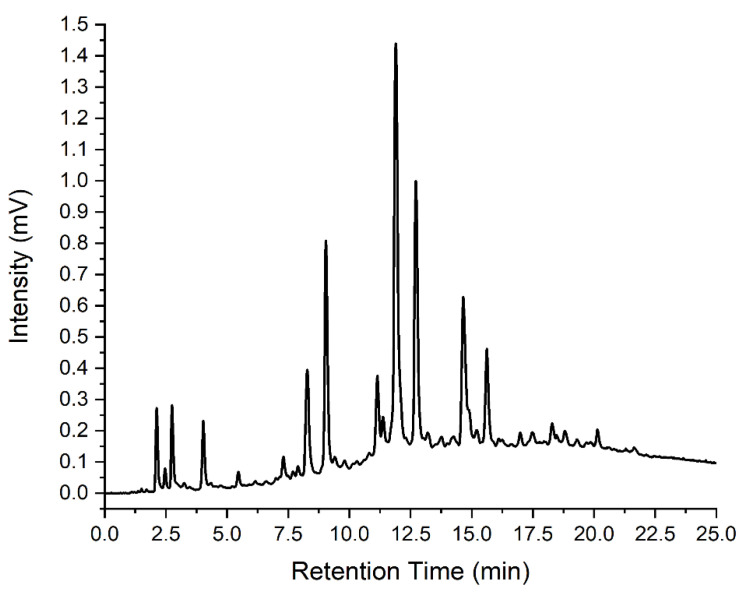
Normalized ICD Chromatographs Response of Ristretto Coffee (λ = 450 nm).

In order to compensate for differences in the mass of coffee from capsule to capsule and the qualitative aspect of coffee preparation (the coffee machine does not reproducibly deliver 30.00 mL shots, for example), the chromatographic response was normalized to the height of an indicator peak that was present in all coffee samples. The elution time of this peak was 12.46 min. This was not the most intense peak in the chromatographic profiles of the coffees tested here; hence, there were instances where the normalized peak heights resulted in values greater than unity. The advantage, however, of using the peak at 12.46 min was that it was always present in concentrations well within the linear dynamic range of the detector. This meant that the coffee samples could be used as prepared, without further dilution, and all normalization that followed was linear. Subsequent to the normalization process, the normalized peak height of each indicator was used to prepare a library of normalized peak heights for each sample of coffee tested. An example of the normalized chromatographic response of the Ristretto sample is shown in Figure 5.

#### 3.3.3. Data Analysis

**Figure 6:**
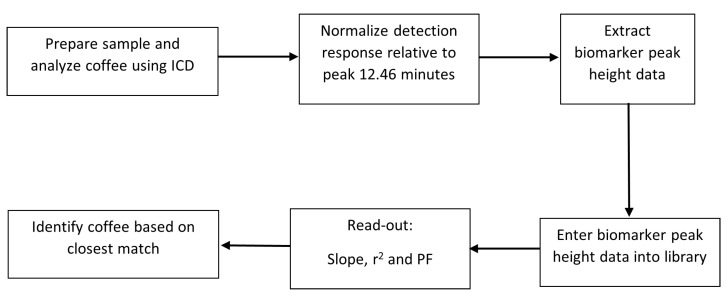
Flow diagram of the 6 steps in the process of analysis.

The identification of an unknown coffee sample is subsequently based on the analysis of the coffee using the ICD process, extraction of the normalized peak height data for the 15 indicator compounds, and then searching the library for a match to the coffee sample from within the library. This process is outlined in Figure 6.

We employed three simple metrics in relation to the library-collated normalized peak-height data obtained through the ICD analysis process. These metrics would lead to being able to identify coffee relative to the library data. Two of these are based on correlation plots between coffee ‘A’ and coffee ‘B’, where ‘A’ and ’B’ refer to two coffee samples that are being compared, for example, Ristretto and Profondo. Specifically, measurement of (1) the slope of the correlation between ‘A’ and ‘B’, where a unit slope (in consideration also of metric 2) is indicative of a perfect match between the sample coffee (‘B’) and the reference coffee (‘A’), and (2) the r^2^ value for the best linear fit. The slope and the r^2^ values should be viewed collectively. It is entirely possible that the plot of the indicator peak height of coffee ‘A’ versus coffee ‘B’ yields a near-perfect linear unit slope with a low r^2^ value. This would suggest data scattering uniformly around the ‘line of best fit’, but with low correlation, it is an unlikely match. Likewise, a unit r^2^ value could be obtained with a nonunit slope. This would suggest that the coffees are similar in characteristics, but not identical; rather, they are scalable entities of each other. A third metric was used, which assessed the relative change in peak height of two key indicators, whose concentrations were deemed to be predictably variable in the differing coffees. Specifically, we applied a series of power functions (PFs) to the normalized chromatographic response for each coffee, such that the ratio of the indicator ‘6’ to indicator ‘7’ was 0.1. It was found that the ratio between the peaks at 8.16 (indicator ‘6’) and 8.85 (indicator ‘7’) minutes was particularly indicative of, and unique to, the specific coffee sample, and, as such, these are the peaks utilized in the power function algorithm. The power function that was required to achieve the output ratio of 0.1 was then indicative of the specific coffee sample. The match of the unknown sample coffee to a library coffee sample is then based upon the closeness of matching to these three metrics. The data in Table 3, Table 4 and Table 5 detail the relationships between each of the three metrics for the 30 library coffee samples. 

All data have been processed and analyzed through Microsoft Excel to demonstrate the simplicity of these methods and that the utilization of more complex programming software packages or tailor-made programs to complete this analysis is not essential. 

#### 3.3.4. Testing the Library Matching

**Figure 7:**
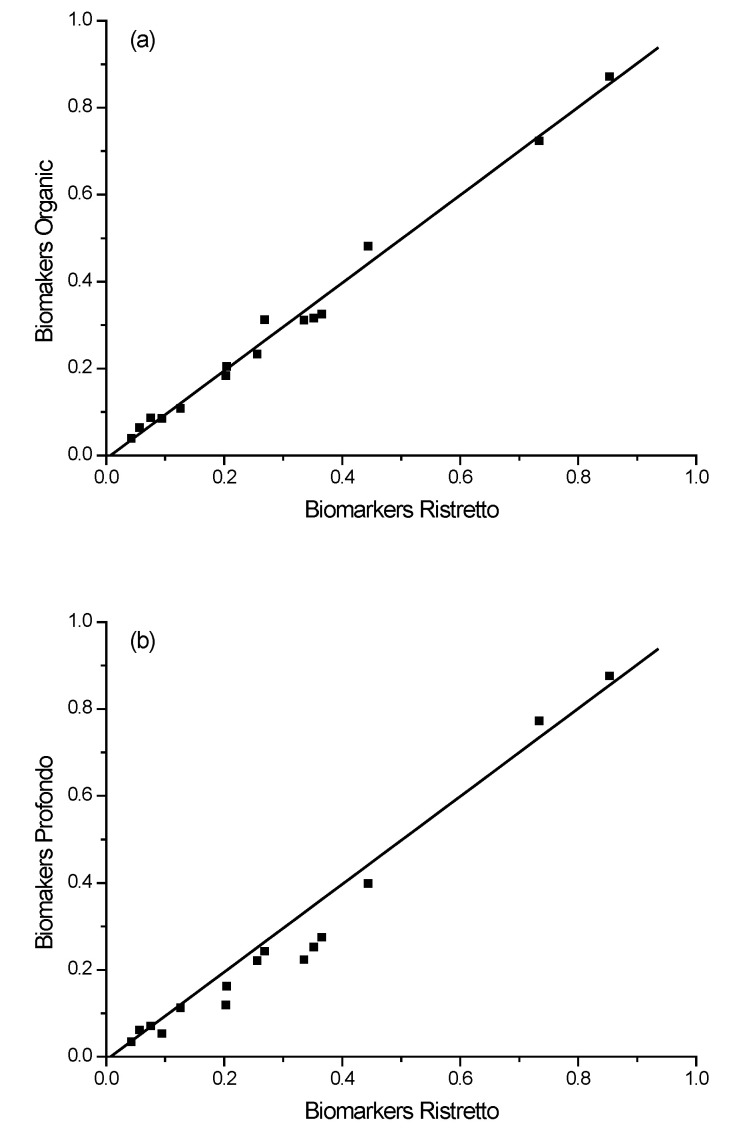

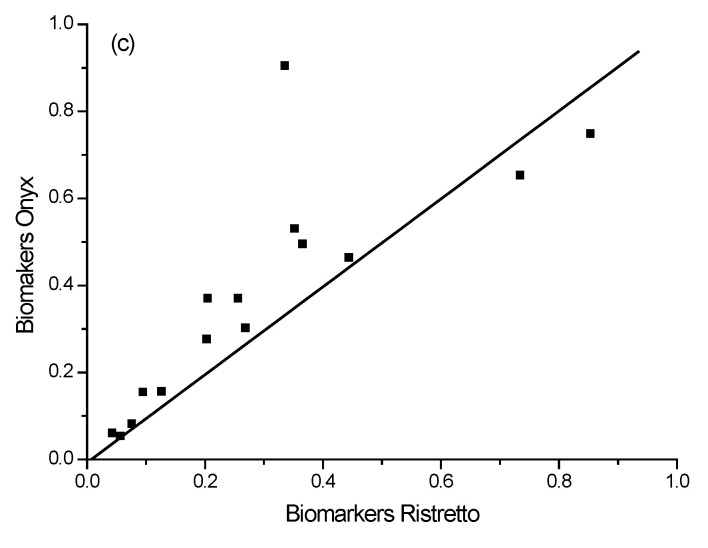
Comparative plots of normalized peak heights for the 15 indicators: (**a**) Organic Ristretto versus Ristretto—both L’Or brand, (**b**) Profondo versus Ristretto—both L’Or brand, and (**c**) Onyx 12 versus Ristretto—both L’Or brand.

After having extracted peak heights from the normalized chromatograms of each coffee, a correlation plot is made between any set of coffees for the purpose of identification, i.e., the unknown sample is compared to a library of data we have collected based on these 30 coffee samples. The plot in Figure 7a illustrates the relationship between the indicators in the L’Or Ristretto coffee relative to the L’Or Organic Ristretto, as an example. These two coffees are virtually indistinguishable based on these correlation plots, which would be expected given they are both ‘Ristretto’ flavors from L’Or and the principal difference between the two is that one is organically grown. The slope of the correlation plot was 0.9988 and the r^2^ value was 0.9971 (see Table 3A and Table 4A, respectively). Both are close to unity, suggesting a match. Next, the power function required for the ratio of the indicator peaks ‘I’ to ‘j’ to reach a value of 0.1 was 2.9381 for the Ristretto and 2.7358 for the Organic Ristretto (Table 5A). The differing power function value suggests that the samples of coffee may not be the same. The power function algorithm was designed such that it exaggerates minute differences in the data sets, explaining this variation between two near-identical coffee samples. Still, two matched metrics and one indifferent indicates a possible match for the two coffees. It should be noted, however, that the ability to distinguish between these two near-identical coffees would always be very problematic.

Further exploring the validity of the chemical signature process, we demonstrate here the comparison between L’Or Ristretto and L’Or Profondo (Figure 7b). The metrics derived from the chromatographic data of each sample of coffee yields a correlation slope value of 0.9492 (nonunit), an r^2^ value of 0.9757 (nonunit), and the power function—Ristretto being 2.9370 and Profondo being 1.8529. Each of these three metrics are different, especially the power function; hence, the library is able to distinguish the difference between these coffees (being a nonmatch).

As another example, for Ristretto, relative to Onyx 12 (Figure 7c), the slope = 0.7388, r^2^ = 0.6312, and the power function—Ristretto = 2.9370 and Onyx 12 = −7.0712. Each of these three metrics are very different; hence, the process described above provides a distinction between the coffee samples.

#### 3.3.5. Unknown Sample Assignment to Library Data

Ultimately, the true test to validate the accuracy of the chemical signature is to be able to identify unknown coffees using the library data. In this study, this was achieved by one member of the team selecting five coffees for analysis; the identity of all five coffees remained unknown to the other team members. These unknown coffee samples were selected as randomly as possible prior to the analysis of any data, hence eliminating the possibility that we have unconsciously selected ‘easy to identify’ coffee samples. After the chromatographic analysis, the normalized peak-height information for the 15 indicators was extracted from the chromatographic separations, and this data was matched to the library data. Following, the best matches for these coffees were ranked, and it was determined that two samples were unequivocally identified as a direct match to just a single library coffee, while the other three were matched to two possible outcomes. In each case, the ranking of the three metrics by the analyst was such that the outcome was preferenced towards the correct coffee match, albeit this was a difficult assignment. In two of these three unknown coffees, the reduction in the uncertainty of the identity of the coffee was a match to another coffee from within the same brand. The result of this analysis is shown in Table 6. A second match indicative of similarities between the capsules, which may be the result of relabeling manufactured products sourced from the same supplier.

## 4. Conclusions

The coffee industry is large, widespread, and very lucrative. There is a need to protect the integrity of coffee products from potential counterfeiters, as it is an easy product to adulterate, and global economic pressures are increasing the number of occurrences of fraud. The spectral fingerprint of a coffee can be used as a unique identifier; however, it is easy to both read and mimic. Utilization of an ICD process involving an antioxidant assay provides a method of utilizing the fingerprint while restricting who can access it. In this paper, we proposed that the construction of a data library would facilitate a means of verifying coffee products by comparison between a genuine product and a coffee in question. We noted that coffees can be characterized by the profile of 15 antioxidant indicators common to most coffees, but which varied in detection response from coffee to coffee. Through the observation of the indicator correlation slope, r^2^ value, and peak-height ratio power function coefficient, it would be possible to classify a coffee by comparison to one within the data library. In this study, a data library was constructed with 30 coffee capsules from mostly local markets, with the intent to investigate how effective the proposed classification method would be. Of five unknowns, two were classified correctly, the others were narrowed down as being one of two possible coffees, and one of these unknowns was matched to a coffee of the same brand. From these results, we conclude that the developed method holds promise, especially given the simplicity and speed of the assay. A single coffee sample could be profiled in around 25 min and matched almost immediately to a library source. The technique was adequately reliable and accurate in its matching capabilities, such that even replication of the assay was not required. However, expansion of the data library is required to reveal its true classification power.

## Figures and Tables

**Table 1:** Characteristics and Description of Coffees tested.

Brand/Flavor	Type of Bean	Flavor Intensity	Description	Mass of Coffee Actual/g
L’Or Ristretto	Unknown, made in France with coffee from multiple origins	11	Notes of ginger and cardamom.	5.586
L’Or Organic Ristretto	Unknown	11	Notes of ginger and cardamom.	5.798
L’Or Profondo	Unknown	8	Intense and spicy aromas, roasted almonds, and licorice.	5.906
L’Or Colombia	Unknown		Smooth and sweet blend, notes of citrus fruit.	5.464
L’Or Papua New Guinea	Unknown, single-origin blend from Papua New Guinea	7	Vibrant, fruity taste notes and deeper roasted notes.	5.473
L’Or Sontuoso	Unknown	8	Notes of crème brulée, nutty marzipan, and sweet cream.	5.552
L’Or Arabica Catuai	Brazilian coffee, grown inside a yellow coffee cherry. Single varietal	7	Zesty, sweet, and roasty.	5.516
L’Or Guatemala	Unknown	7	Mild-roasted flavor with light and zesty notes.	5.627
L’Or Onyx 12	Unknown	12	Dark roasted, with spicy notes.	5.952
L’Or Or Absolu	Unknown	9	Woody notes and reserved sweetness. Intricate balance of delicate sweet caramel and complementing spice.	5.414
L’Or Ultimo	No Information	13	Caramel fruitiness with deeply roasted notes.	5.741
Starbucks House	Blend of fine Latin American beans 100% Arabica	8	Balanced tastes of nuts and cocoa.	5.905
Starbucks Roast	Blend of Latin American and Asia/Pacific coffees	11	Rich and caramelly sweetness.	5.736
Starbucks Colombia	Single origin 100% Arabica	7	Round body, juicy taste, and signature nutty finish.	5.870
Woolworths Intense	Unknown	12	Rich and full-bodied dark roast.	5.687
Woolworths Strong	Unknown	10	Full-bodied dark roast.	5.766
Woolworths Decaf	Arabica	6	Sweet, well-balanced espresso.	5.709
Vittoria Ristretto	100% Arabica blend	13	Sweet, nutty, and butterscotch.	5.131
Vittoria Espresso	100% Arabica blend from Central and South America		A dark-roasted coffee blend featuring a rich, full and flavorsome taste.	5.174
Starbucks Sumatra	Single-origin from Indonesia 100% Arabica	10	Spicy and full-bodied, with a herbal taste.	5.797
Starbucks Decaf Espresso Roast	Blend from Asia and Latin America 100% Arabica	11	Rich and caramelly.	5.900
Starbucks Café Verona	100% Arabica	10	Roasty sweet with dark cocoa notes.	5.780
Moccona Long Black	Unknown	8	Fruity notes with coffee bitters and chocolate sweetness.	5.468
Daley St Med Roast	Colombian and Kenyan Arabica Beans	8	Notes of chocolate, licorice, and toffee.	5.252
Daley St Intense Roast	Colombian Arabica and Vietnamese Robusta Beans	12	Notes of treacle, malt, and prune.	5.307
Vittoria Black Valley	100% Arabica from Brazil, Colombia, and Honduras	15	Syrupy and full-flavored. Smokey.	5.245
Vittoria Inca Peru	100% Arabica from Brazil, Costa Rica, and the Cajamarca, Chirinos, and Cuzco regions of Peru	15	Maple syrup, roasted peanuts, and blossoms.	5.192
Moccona Barista Reserve Long Black	100% ground coffee	8	Fruity notes, coffee bitters, and chocolatey sweetness.	5.436
Ari coffee, Nan (local Thailand brand)	100% Arabica from Nan, Thailand	N/A	Medium—dark roasted	5.300
Ari coffee, Chum Phon (local Thailand brand)	100% Arabica from Chum Phon, Thailand	N/A	Medium—dark roasted	5.133
Ari coffee, Doi Pang Khon (local Thailand brand)	100% Arabica from Chiang Rai, Thailand	N/A	Medium—dark roasted	5.690
Ari coffee, Doi Chang (local Thailand brand)	100% Arabica from Chiang Mai, Thailand	N/A	Medium—dark roasted	4.780

**Table 2:** Retention time of selected indicators. Bolded is the reference indicator used for normalization.

Indicator	Retention Time (Minutes)
1	2.08
2	2.41
3	2.67
4	3.95
5	5.37
6	8.16
7	8.85
8	10.88
9	11.12
10	12.46
11	14.44
12	15.45
13	16.76
14	18.19
15	20.12

**Table 3:** A selection of the coffee similarity data according to slope for (**A**) the L’Or brand coffees and (**B**) a selection of mixed brand coffees.

**(A)**											
	**Ristretto**	**Organic R**	**Profondo**	**Columbia**	**Papua**	**Ultimo**	**Sontuoso**	**Arabica C**	**Or Ab-solu**	**Gua-temala**	**Onyx 12**
Ristretto	1.0000										
Organic R	0.9983	1.0000									
Profondo	0.9492	0.9500	1.0000								
Columbia	0.9687	0.9700	1.0162	1.0000							
Papua	0.9579	0.9615	1.0046	0.9864	1.0000						
Ultimo	0.9848	0.9759	0.9882	0.9786	0.9794	1.0000					
Sontuoso	0.9532	0.9560	1.0008	0.9813	0.9943	0.8957	1.0000				
Arabica C	0.9178	0.9203	0.9748	0.9529	0.9643	0.8544	0.9708	1.0000			
Or Absolu	0.9862	0.9831	1.0095	0.9848	1.0000	0.9511	1.0062	1.0223	1.0000		
Guatemala	0.8802	0.8863	0.9408	0.9225	0.9344	0.8095	0.9388	0.9677	0.8619	1.0000	
Onyx 12	0.7388	0.7202	0.6881	0.6782	0.6756	0.8086	0.6779	0.6658	0.7738	0.6333	1.0000
**(B)**											
	**Ristretto**	**SB House**	**SB Roast**	**SB Col**	**W Int**	**W Strong**	**Decaf**	**Ari, Nan**	**Ari, Chum Phon**	**Ari, Doi Pang Khon**	
Ristretto	1.0000										
SB House	0.9225	1.0000									
SB Roast	0.4429	0.6247	1.0000								
SB Col	0.9568	1.0111	1.1295	1.0000							
W Int	0.9648	0.8635	0.6641	0.8665	1.0000						
W Strong	0.9263	0.8093	0.5765	0.8141	0.9688	1.0000					
Decaf	0.8320	0.6838	0.4044	0.6974	0.8749	0.9076	1.0000				
Ari, Nan	0.0641	0.1764	0.3856	0.1520	0.0419	0.0246	−0.0192	1.0000			
Ari, Chum Phon	0.6792	0.7542	0.8381	0.7164	0.7122	0.7123	0.7041	1.5349	1.0000		
Ari, Doi Pang Khon	0.7335	0.5933	0.3451	0.6082	0.7765	0.7907	0.8814	0.0158	0.7014	1.0000	

**Table 4:** A selection of the coffee similarity data according to R^2^ for (**A**) the L’Or brand coffees and (**B**) a selection of mixed brand coffees.

**(A)**											
	**Ristretto**	**Organic R**	**Profondo**	**Columbia**	**Papua**	**Ultimo**	**Sontuoso**	**Arabica C**	**Or Absolu**	**Guatemala**	**Onyx 12**
Ristretto	1.0000										
Organic R	0.9971	1.0000									
Profondo	0.9757	0.9767	1.0000								
Columbia	0.9792	0.9814	0.9951	1.0000							
Papua	0.9788	0.9858	0.9942	0.9946	1.0000						
Ultimo	0.9509	0.9333	0.8841	0.8997	0.8816	1.0000					
Sontuoso	0.9780	0.9832	0.9956	0.9932	0.9976	0.8807	1.0000				
Arabica C	0.9554	0.9602	0.9954	0.9871	0.9888	0.8445	0.9932	1.0000			
Or Absolu	0.9795	0.9730	0.9479	0.9361	0.9441	0.9294	0.9474	0.9280	1.0000		
Guatemala	0.9302	0.9427	0.9813	0.9791	0.9826	0.8025	0.9832	0.9913	0.8854	1.0000	
Onyx 12	0.6312	0.5994	0.5056	0.5097	0.4948	0.7712	0.4936	0.4518	0.6874	0.3863	1.0000
**(B)**											
	**Ristretto**	**SB House**	**SB Roast**	**SB Col**	**W Int**	**W Strong**	**Decaf**	**Ari, Nan**	**Ari, Chum Phon**	**Ari, Doi Pang Khon**	
Ristretto	1.0000										
SB House	0.8569	1.0000									
SB Roast	0.3653	0.7219	1.0000								
SB Col	0.8941	0.9915	0.6690	1.0000							
W Int	0.9597	0.7635	0.2442	0.7927	1.0000						
W Strong	0.9357	0.7093	0.1946	0.7402	0.9929	1.0000					
Decaf	0.8971	0.6017	0.1138	0.6455	0.9621	0.9787	1.0000				
Ari, Nan	0.0363	0.2726	0.7044	0.2088	0.0150	0.0049	0.0025	1.0000			
Ari, Chum Phon	0.6286	0.7696	0.5140	0.7161	0.6703	0.6339	0.5213	0.3638	1.0000		
Ari, Doi Pang Khon	0.7284	0.4734	0.0866	0.5129	0.7918	0.7762	0.8117	0.0000	0.4889	1.0000	

**Table 5:** Power functions for each coffee for (**A**) L’Or Brand Coffees and (**B**) mixed brand coffees.

**(A)**	
**Coffee**	**Power Function**
Ristretto	2.9370
Organic Ristretto	2.7358
Profondo	1.8529
Columbia	1.8601
Papua	1.8332
Ultimo	4.5438
Sontuoso	1.8265
Arabica Catuai	1.5687
Or Absolu	4.0843
Guatemala	1.2538
Onyx 12	−7.0712
**(B)**	
**Coffee**	**Power Function**
Starbucks House	114.0513
Starbucks Roast	−3.0854
Woolworths Intense	2.3636
Woolworths Strong	1.9145
Woolworths Decaf	1.0272
Vittoria Ristretto	1.0670
Vittoria Espresso	1.5741
Sumatra	−3.0136
Decaf Espresso Roast	−3.1050
Café Verona	−9.2563
Long Black	1.8319
Daley St Med Roast	1.1996
Daley St inter Roast	2.2443
Black Valley	1.1064
Inca Peru	1.1441
Starbucks Columbia	14.3692
Ari, Nan	−1.1064
Ari, Chum Phon	−10.5532
Ari, Doi Pang Khon	1.3555

**Table 6:** Testing outcome and unknown match to library standard coffees.

	Coffee				
Metric	Unknown 1	Unknown 2	Unknown 3	Unknown 4	Unknown 5
**Highest match**	**Unk1:** **Starbucks Columbia**	**Unk2:** **Guatemala**	**Unk3:** **Vittoria Espresso**	**Unk4:** **Profondo**	**Unk5:** **Or Absolu**
Slope	0.9765	0.9999	0.9782	0.9903	0.9711
R^2^	0.9748	0.9887	0.9956	0.9914	0.9879
P.F.	14.3692	1.2538	1.5741	1.8529	4.0843
**Second Highest Match**	**No** **Second Match**	**Unk2:** **Inca Peru**	**Unk3:** **Arabica Catuai**	**Unk4:** **Long Black**	**No** **Second Match**
Slope		1.0043	1.0022	1.0007	
R^2^		0.9958	0.9831	0.9899	
P.F.		1.1441	1.5687	1.8319	

## Data Availability

Data available upon request to the authors.
